# Cyberbullying victimisation and internalising and externalising problems among adolescents: the moderating role of parent–child relationship and child's sex

**DOI:** 10.1017/S2045796018000653

**Published:** 2018-11-13

**Authors:** H. Sampasa-Kanyinga, K. Lalande, I. Colman

**Affiliations:** 1 School of Epidemiology and Public Health, University of Ottawa, Ottawa, Ontario, Canada; 2 The Child, Adolescent, and Family Centre of Ottawa, Ottawa, Ontario, Canada; 3 Centre for Psychological Services and Research, School of Psychology, Faculty of Social Sciences, University of Ottawa, Ottawa, Ontario, Canada

**Keywords:** Bullying, cyberbullying, psychological distress, substance use, suicidality

## Abstract

**Aims:**

Previous research has found links between cyberbullying victimisation and internalising and externalising problems among adolescents. However, little is known about the factors that might moderate these relationships. Thus, the present study examined the relationships between cyberbullying victimisation and psychological distress, suicidality, self-rated poor mental health and substance use among adolescents, and tested whether parent–child relationship and child's sex would moderate these relationships.

**Methods:**

Self-report data on experiences of cyberbullying victimisation, self-rated poor mental health, psychological distress, suicidality and substance use were derived from the 2013 Ontario Student Drug Use and Health Survey, a province-wide school-based survey of students in grades 7 through 12 aged 11–20 years (*N* = 5478). Logistic regression models adjusted for age, sex, ethnicity, subjective socioeconomic status and involvement in physical fighting, bullying victimisation and perpetration at school.

**Results:**

Cyberbullying victimisation was associated with self-rated poor mental health (adjusted odds ratio (OR) 2.15; 95% confidence interval (CI) 1.64–2.81), psychological distress (OR 2.41; 95% CI 1.90–3.06), suicidal ideation (OR 2.38; 95% CI 1.83–3.08) and attempts (OR 2.07; 95% CI 1.27–3.38), smoking tobacco cigarette (OR 1.96; 95% CI 1.45–2.65), cannabis use (OR 1.82; 95% CI 1.32–2.51), and binge drinking (OR 1.44; 95% CI 1.03–2.02). The association between cyberbullying victimisation and psychological distress was modified by parent–child relationship and child's sex (three-way interaction term *p* < 0.05). The association between cyberbullying victimisation and psychological distress was much stronger among boys who have a negative relationship with their parents.

**Conclusions:**

Findings suggest that cyberbullying victimisation is strongly associated with psychological distress in most adolescents with the exception of males who get along well with their parents. Further research using a longitudinal design is necessary to disentangle the interrelationship among child's sex, parent–child relationship, cyberbullying victimisation and mental health outcomes among adolescents in order to improve ongoing mental health prevention efforts.

## Introduction

Cyberbullying is a serious public-health problem worldwide that has devastating effects for the victim, family, school and the wider community. Cyberbullying is often defined as the use of email, cell phones, text messages and the Internet to threaten, harass, embarrass or socially exclude (Hinduja and Patchin, 2009). With the rapid advances in information and communication technologies, cyberbullying has become increasingly widespread among adolescents (Kraft, 2006; Schneider *et al*., 2015; Selkie *et al*., 2016). About one in five adolescents has experienced cyberbullying victimisation in the past year (Schneider *et al*., 2012; Elgar *et al*., 2014; Sampasa-Kanyinga *et al*., 2014). Several features distinguish cyberbullying from traditional bullying, including, but not limited to, the pervasiveness of victimisation – as it can follow a victim everywhere for 24 h a day and 7 days a week, the anonymity afforded to perpetrators, the limitless potential audience consisting of bystanders and observers, the inability for victims to have any control over acts of cyberbullying, the inability of perpetrators of cyberbullying to fully consider the depth of the consequences of their actions towards their victims, and the reluctance of the victims to report their experience of victimisation to their parents (Li, 2006; Dehue *et al*., 2008; Slonje and Smith, 2008; Kowalski *et al*., 2014). As a result, cyberbullying could result in more devastating effects for the victims than those of school bullying (Campbell *et al*., 2012). For example, a meta-analysis of 34 studies found that cyberbullying was more strongly associated with suicidal ideation than was traditional bullying (van Geel *et al*., 2014). It is well known that victims of cyberbullying experience internalising (e.g., anxiety, depression and suicidal ideation and attempts) and externalising (e.g., violence and substance abuse) problems (Sampasa-Kanyinga *et al*., 2014; Tsitsika *et al*., 2015; Fisher *et al*., 2016). Novel research is therefore necessary to identify possible factors that could buffer the risk of negative outcomes related to these threats. Identification of these factors can inform the development of effective interventions to reduce the risk of mental health problems related to cyberbullying victimisation.

One factor that has received little attention is the role of the parent–child relationship, despite accumulating evidence of protective effects of parental support against the effects of bullying and mental health outcomes among adolescents (Patten *et al*., 1997; Stice *et al*., 2004; Machmutow *et al*., 2012). Research has shown that a positive parent–child relationship has a buffering effect on adolescent risk taking and negative outcomes (Gribble Patricia *et al*., 2006; O'Brien and Mosco, 2012; Qu *et al*., 2015), such as mental health problems (Stafford *et al*., 2016). For example, Aseltine *et al*. (1998) have shown that high-quality parent–adolescent relationships predict lower levels of adolescent depression (Aseltine *et al*., 1998). Lower rates of parent–child conflict have also been prospectively associated with less externalising symptoms, conduct problems and antisocial behaviours (Burt *et al*., 2006; Klahr *et al*., 2011). According to the stress-buffering model, social support or positive relationships with others, protects against the potentially pathogenic influence of stressful events (Cohen and Wills, 1985). As such, parents could help protect their children from maladjustment by helping them cope with stress (Bowlby, 1988). According to the attachment theory, a sense of secure emotional connection to key individuals, such as parents, caregivers or other adults provides a base for psychological and social development (Bowlby, 2005). Thus, it is plausible that a positive parent–child relationship could buffer the effects of cyberbullying victimisation. However, previous research examining the associations between cyberbullying victimisation and internalising and externalising behaviours has been limited by the use of proxy measures for the parent–child relationship, such as the frequency of family dinner (Elgar *et al*., 2014). Even though the frequency of evening family meals offers an opportunity to family contact and communication – thereby potentially protecting against mental health problems and risk behaviours among adolescents (Fulkerson *et al*., 2006; Elgar *et al*., 2013) – this measure may not capture the true nature of parent–child relationship.

Furthermore, it is unclear whether the possible interrelationships between cyberbullying victimisation, mental health problems and parent–child relationship would vary between males and females. Research suggests that parent–child relationships differentially vary between adolescent males and females. Mother–daughter relationships are generally strongest, whereas father–daughter relationships are not as strong (Steinberg, 1987). Similarly, fathers are usually closer to their sons than daughters (Starrels, 1994). It is possible that the gender differences in the parent–child relationships differentially protect against negative outcomes between males and females. On the other hand, research examining sex differences in experiences of cyberbullying victimisation has reported mixed findings. Some studies have found that females are more likely than males to be victims of cyberbullying (Kowalski and Limber, 2007; Dehue *et al*., 2008; Smith *et al*., 2008; Schneider *et al*., 2012; Elgar *et al*., 2014; Sampasa-Kanyinga *et al*., 2014), whereas others did not find such differences (Li, 2007; Wade and Beran, 2011). Bannink *et al*. (2014) have previously documented the moderating role of sex on the relationship between cyberbullying victimisation and mental health problems in a sample of more than 3000 Dutch secondary school students. They found that cyberbullying victimisation was prospectively associated with mental health problems among females, but not males (Bannink *et al*., 2014). Examining whether the interrelationship between cyberbullying victimisation, internalising and externalising problems and parent–child relationship varies by sex is particularly important because it will help identify at risk groups and inform future mental health intervention and prevention efforts.

In the present study we examined the relationships between cyberbullying victimisation and psychological distress, other mental health outcomes and substance use in adolescent males and females, and tested whether parent–child relationship and sex would moderate these relationships. We hypothesised that cyberbullying victimisation would result in poor mental health and greater substance use behaviour; and that these relationships would be stronger among adolescents who have negative relationship with their parents, particularly females.

## Methods

Data for this study were derived from the 2013 cycle of the Ontario Student Drug Use and Health Survey (OSDUHS), a cross-sectional school-based survey of grade 7–12 Ontario students, aged 11–20 years (Boak *et al*., 2013). The survey employs a stratified (region and school type (i.e., elementary *v.* secondary)), two-stage (school, class) cluster sample design. Within each stratum, schools were selected with probability-proportional-to-size, and within selected schools, classes were selected with equal probability. Completion rates were 94% and 63% for schools and students, respectively, above average for a survey of students that requires active parental consent (Courser *et al*., 2009). To include as many topics as possible in a fixed class period, while minimising the burden on students, the survey used four split ballot modularised questionnaires (forms A and B), depending on school level, in a paper booklet format. Students completed one of two alternately distributed (i.e., A, B, A, B) anonymous, self-administered questionnaires in their classrooms. Both forms had questions on sociodemographic characteristics, parent–child relationship, smoking, binge drinking and cannabis use, while form A exclusively contained mental health, physical fighting and bullying questions. The total sample consisted of 10 272 students drawn from 42 school boards, 198 schools and 671 classes. However, the analyses of the present study are restricted to the random half sample of students (*N* = 5478) who were administered the questionnaire form that contained the mental health and cyberbullying items (i.e., form A). Included participants did not differ from the excluded group for any selected characteristics (i.e., sociodemographic characteristics, parent–child relationship, smoking, binge drinking and cannabis use). Detailed information about the methodology of the 2013 OSDUHS is available on-line (Boak *et al*., 2013). Ethics approval was obtained from the Research Ethics Committees of the Centre for Addiction and Mental Health, St. Michael's Hospital, participating Ontario Public and Catholic school boards, and York University, which administered the surveys. All participants provided their signed assent in addition to parentally signed consent for those under 18 years.

### Independent variable: cyberbullying victimisation

Involvement in school bullying behaviour and victimisation (described below with other covariates) and cyberbullying victimisation in the past 12 months were measured using items that were adapted from the World Health's Organization's Health Behaviour of School-aged Children (HBSC) study (Boak *et al*., 2013). Bullying was defined as repeatedly being teased by one or more people, being hurt or upset or being left out of things on purpose (Boak *et al*., 2013). Cyberbullying victimisation was measured by the following question: ‘In the last 12 months, how many times did other people bully or pick on you through the Internet?’ Responses included do not use internet, never, once, two to three times and four or more times. A dichotomous measure was created to represent ‘never been cyberbullied’ and ‘been cyberbullied at least once’ in the last 12 months.

### Primary outcomes

#### Self-reported mental health

Self-rated mental health was assessed by the following question: ‘How would you rate your mental or emotional health?’ The answer options were: ‘poor’, ‘fair’, ‘good’, ‘very good’ or ‘excellent’. Responses of ‘fair’ or ‘poor’ were collapsed to indicate ‘poor mental health’ (Sampasa-Kanyinga and Lewis, 2015).

#### Psychological distress

The Kessler Psychological Distress Scale (K10) was used to measure symptoms of depression and anxiety occurring over the most recent 4-week period (Kessler *et al*., 2002; Kessler *et al*., 2003), using the following items: in the past 4 weeks, about how often did you feel (1) tired out for no good reason; (2) nervous; (3) so nervous that nothing could calm you down; (4) hopeless; (5) restless or fidgety; (6) so restless you could not sit still; (7) depressed; (8) that everything was an effort; (9) so sad that nothing could cheer you up; (10) worthless? Each of the ten items had five response categories including ‘none of the time’, ‘a little of the time’, ‘some of the time’, ‘most of the time’ and ‘all of the time’. Responses are scored on a five-point Likert scale and summed to generate a total score ranged from 10 to 50, with higher scores indicating greater psychological distress. High psychological distress was defined as having a score of ⩾22, while a score of <22 indicated low psychological distress (Sampasa-Kanyinga and Hamilton, 2015*a*; Sampasa-Kanyinga and Lewis, 2015). The internal reliability coefficient for the K-10 in this study was Cronbach's *α* = 0.92.

#### Suicidal behaviour

Suicidal ideation was measured by the following item: ‘During the last 12 months, did you ever seriously consider attempting suicide?’ and suicide attempts were measured by the following item: ‘In the last 12 months, did you actually attempt suicide?’ Response options for both suicidal ideation and attempts were yes and no. Both questions are from the Centers for Disease Control and Prevention (CDC)’s Youth Risk Behaviour Survey and have demonstrated good reliability and validity among students (May and Klonsky, 2011).

### Secondary outcomes: substance use

Tobacco cigarette smoking and cannabis use were measured with the following two questions: ‘In the last 12 months, how often did you smoke cigarettes?’ and ‘In the last 12 months, how often did you use cannabis (e.g., ‘marijuana’)?’ Responses were binary coded as ‘used at least once’ or ‘did not use’. Binge drinking was measured through question asking how often students had five or more drinks of alcohol on the same occasion (i.e., binge drinking) during the past 4 weeks. Response options were yes and no.

### Potential moderator: parent–child relationship

Parent–child relationship was assessed using a combination of the following two items: (1) How well would you say you are getting along with your mother? Response options included:

‘I am getting along very well with my mother’, ‘I am getting along OK with my mother’,

‘I am not getting along well with my mother’ and ‘no mother’. (2) How well would you say you are getting along with your father? Response options included: ‘I am getting along very well with my father’, ‘I am getting along OK with my father’, ‘I am not getting along well with my father’ and ‘no father’. Responses were binary coded as ‘getting along very well or OK with mother or father’ or ‘Do not get along well with mother or father’. Getting along very well or OK with at least one parent were collapsed to represent ‘positive parent–child relationship’ contrasting with negative parent–child relationship (i.e., not getting along well with both parents). A relatively small number of respondents reported no mother (*n*  =  44) or no father (*n*  =  201) and have been included among those who do not get along well with parents because conceptually, children who have no parents and those who have negative ‘parent–child relationship’ are both deprived from the potential benefit of a positive parent–child relationship.

### Covariates

Sociodemographic characteristics included age, sex, grade, ethnicity and subjective sociodemographic status (SES). Grades 7 and 8 were grouped as ‘middle school’ and grades 9 through 12 were collapsed to represent ‘high school’. The youth version of the MacArthur Scale of Subjective Social Status (Goodman *et al*., 2001) was slightly modified to assess the family's place within society. A ladder of ten rungs was drawn and respondents were asked to place an ‘X’ on the rung on which they feel they stand based on SES indicators, including money, education and jobs. A dichotomous measure was constructed to represent low (<7) and high (⩾7) subjective SES, wherein low scores represent those below the mean (Sampasa-Kanyinga and Hamilton, 2015*b*). Involvement in physical fighting was included as a covariate to control for the confounding effects of aggressive behaviour associated with both mental health problems and cyberbullying victimisation (Loeber *et al*., 2000; Elgar *et al*., 2014). Participants were asked how often they got into a physical fight at school during the last 12 months. Response options included the following count scale: never, once, two or three times, four or five times, six or seven times, eight or nine times, 10 or 11 times, 12 or more times. The last three response options were collapsed to represent eight or more times. The measure was treated as scale variables ranging from 1 to 6. School bullying victimisation and perpetration were also included as covariates because they commonly co-occur with cyberbullying victimisation (Gradinger *et al*., 2009; Waasdorp and Bradshaw, 2015; Sampasa-Kanyinga, 2017). School bullying involvement was measured using two items. (1) Students were asked if they were bullied at school since September. Response options included ‘was not bullied at school since September’, ‘daily or almost daily’, ‘about once a week’, ‘about once a month’ and ‘less than once a month’. (2) Students were also asked to indicate how often they bullied other students since September. Response options included ‘did not bully at school since September’, ‘daily or almost daily’, ‘about once a week’, ‘about once a month’ and ‘less than once a month’. Both measures were treated as scale variables ranging from 1 to 5.

### Statistical analyses

The Taylor Series Linearisation method was used to estimate variances from our stratified and clustered survey data. The estimation model was based on a design with 20 strata (region by school level) and 198 primary sampling units (schools). We used cross-tabulations to examine bivariate associations of cyberbullying victimisation and parent–child relationship with mental health (self-rated mental health, psychological distress, suicidal ideation and attempts) and substance use (smoking tobacco cigarettes, cannabis use and binge drinking) outcomes. Data were compared using a Pearson *χ*^2^ adjusted for the survey design and transformed into an *F*-statistic. Logistic regression analyses were performed to examine the associations between cyberbullying victimisation and outcome variables of self-rated poor mental health, psychological distress, suicidal ideation and attempts, smoking tobacco cigarette, cannabis use and binge drinking. Models were unadjusted (model 1) and adjusted for age, sex, subjective socioeconomic status and involvement in physical fighting, bullying victimisation and perpetration at school (model 2). In order to test whether the associations between cyberbullying victimisation and all the outcomes vary by parent–child relationship or child's sex, two-way interactions were examined in separate models (models 3 and 4). Subsequent analyses examining the association between cyberbullying victimisation and psychological distress was stratified by child's sex and parent–child relationship because the three-way interaction between cyberbullying victimisation, sex and parent–child relationship was significant. Missing data were handled through complete case analyses for unadjusted and adjusted models per outcomes. All data were analysed with STATA (version 13.0, Stata Corp., College Station, Texas) with a significant *α* threshold of 5%.

## Results

Table 1 shows the characteristics of the study sample. Nearly half of the sample was female (47.8%), and the majority of students (75.5%) were in high school (i.e., grades 9–12), and had high perceived family status (70.9%). Nearly three-in-five students identified themselves as White (59.7%).

**Table 1.** Descriptive characteristics of the study sample

	*n*^a^	%	95% CI
Total	5478	100	
Age			
Mean (s.d.) in years	15.19 (1.82)		15.03–15.35
Missing	0	0	
Sex			
Males	2469	52.17	49.70–54.62
Females	3009	47.83	45.38–50.30
Missing	0	0	
Grade			
7	1126	11.92	9.73–14.52
8	1088	12.55	10.16–15.40
9	815	16.82	15.14–18.64
10	816	17.13	15.56–18.83
11	837	17.76	16.26–19.37
12	796	23.82	21.68–26.10
Missing	0	0	
Subjective socioeconomic status			
Low	1470	29.09	26.68–31.63
High	4008	70.91	68.37–73.32
Missing	0	0	
Ethnicity			
White	3205	59.73	55.18–64.12
Black	300	5.43	4.42–6.65
East/SES Asian	510	9.60	7.61–12.05
South Asian	554	9.78	7.54–12.61
Other	877	15.12	12.94–17.59
Missing	32	0.33	0.18–0.62
Physical fighting			
Mean (s.d.)	1.18 (0.64)		1.16–1.21
Missing	201	3.67	
School bullying victimisation			
Mean (s.d.)	1.47 (0.98)		1.42–1.52
Missing	105	1.92	
School bullying perpetration			
Mean (s.d.)	1.25 (0.69)		1.22–1.29
Missing	104	1.90	

a Data are shown as count unless otherwise indicated.

Table 2 presents the prevalence of cyberbullying victimisation, parent–child relationship and mental health and substance use outcomes by sex. Overall, 18.7% of students reported experience of cyberbullying victimisation, 14.6% reported a negative relationship with their parents and 15.1% of students self-perceived poor mental health. Just over a quarter reported psychological distress in the past month and cannabis use in the past 12 months, 12.3% and 3.2% of students reported suicidal ideation and attempts, respectively. About 19% of students reported smoking tobacco cigarette in the past 12 months. Females were more likely than males to report cyberbullying victimisation and mental health outcomes, including poor self-rated mental health, psychological distress and suicidal ideation and attempts. Males were more likely to report smoking tobacco cigarette and cannabis use than their female counterparts. There were no sex differences for parent–child relationship and binge drinking.

**Table 2.** Prevalence of cyberbullying victimisation, getting well with parents and mental health and substance use outcomes among adolescents by sex

	Total sample (*N* = 5478)	Males (*N* = 2469)	Females (*N* = 3009)	*p* value^a^
	*n* (%)	*n* (%)	*n* (%)	
Cyberbullying victimisation				
No	4296 (79.37)	2046 (82.0)	2250 (76.5)	<0.001
Yes	1095 (18.65)	370 (15.4)	725 (22.2)	
Missing	87 (1.97)	53 (2.6)	34 (1.3)	
Parent–child relationship				
Positive	4748 (85.26)	2182 (86.6)	2566 (83.8)	0.060
Negative	721 (14.59)	279 (13.1)	442 (16.2)	
Missing	9 (0.16)	8 (0.3)	1 (0.0)	
Self-rated mental health				
Good	4548 (83.18)	2179 (87.7)	2369 (78.3)	<0.001
Poor	811 (15.06)	226 (10.3)	585 (20.2)	
Missing	119 (1.76)	64 (2.0)	55 (1.5)	
Psychological distress				
No	4083 (74.37)	2080 (83.1)	2003 (64.9)	<0.001
Yes	1395 (25.63)	389 (16.9)	1006 (35.1)	
Missing				
Suicidal ideation				
No	4516 (79.36)	2135 (82.3)	2381 (76.2)	<0.001
Yes	718 (12.26)	205 (8.5)	513 (16.3)	
Missing	244 (8.39)	129 (9.2)	115 (7.5)	
Suicidal attempt				
No	5068 (88.61)	2305 (89.2)	2763 (88.0)	0.002
Yes	175 (3.18)	39 (1.8)	136 (4.6)	
Missing	235 (8.21)	125 (9.0)	110 (7.4)	
Smoking tobacco cigarette				
No	4691 (81.03)	2072 (78.4)	2619 (84.0)	0.005
Yes	778 (18.75)	391 (21.4)	387 (15.9)	
Missing	9 (0.22)	6 (0.3)	3 (0.2)	
Binge drinking				
No	4522 (79.26)	2069 (77.45)	2553 (81.24)	0.072
Yes	830 (20.39)	383 (22.07)	447 (18.56)	
Missing	26 (0.34)	17 (0.48)	9 (0.20)	
Cannabis use				
No	4383 (73.22)	1941 (70.3)	2442 (76.4)	0.005
Yes	1095 (26.78)	528 (29.7)	567 (23.6)	
Missing				

a Differences between males and females using a Pearson *χ*^2^ adjusted for the survey design and transformed into an *F*-statistic.

Bivariate associations of cyberbullying victimisation and parent–child relationship with mental health and substance use outcomes are outlined in Table 3. Results showed that victims of cyberbullying were more likely than non-victims to report poor self-rated mental health, psychological distress, suicidal ideation and attempts, smoking tobacco cigarette and cannabis use, but not binge drinking. However, those who reported a negative parent–child relationship were more likely than those who reported a positive relationship to report poor self-rated mental health, psychological distress, suicidal ideation and attempts, smoking tobacco cigarette, cannabis use and binge drinking.

**Table 3.** Bivariate associations between cyberbullying victimisation and getting along with parents with mental health and substance use outcomes

	Poor self-rated mental health	Psychological distress	Suicidal ideation	Suicide attempt	Smoking tobacco cigarette	Cannabis use	Binge drinking
	% (95% CI)	% (95% CI)	% (95% CI)	% (95% CI)	% (95% CI)	% (95% CI)	% (95% CI)
Cyberbullying victimisation							
No	11.83 (10.01–13.94)	20.5 (18.32–22.88)	9.54 (8.23–11.04)	2.18 (1.59–2.97)	16.63 (14.04–19.59)	24.81 (21.81–28.08)	19.64 (17.27–27.25)
Yes	30.68 (27.15–34.44)	48.36 (43.79–52.95)	29.61 (26.05–33.44)	9.03 (6.41–12.57)	27.49 (22.96–32.54)	34.34 (30.07–38.88)	22.75 (18.39–27.78)
Parent–child relationship							
Positive	11.88 (10.26–13.72)	21.99 (20.07–24.02)	10.16 (8.7–11.84)	2.19 (1.66–2.88)	16.39 (14.03–19.05)	24.48 (21.65–27.54)	18.85 (16.54–21.40)
Negative	35.53 (29.63–41.91)	46.63 (40.39–52.98)	32.17 (26.3–38.65)	10.86 (7.99–14.59)	32.99 (26.44–40.28)	40.49 (33.51–47.89)	30.07 (25.23–35.40)

All associations are significant at *p* value <0.001 except for binge drinking which was not significant (*p*  =  0.158).

CI, confidence interval.

Table 4 presents univariable and multivariable logistic regression analyses examining the relationships between cyberbullying victimisation and mental health and substance use outcomes. After adjusting for important covariates (model 2), cyberbullying victimisation was associated with self-rated poor mental health (odds ratio (OR) 2.15; 95% confidence interval (CI) 1.64–2.81), psychological distress (OR 2.41; 95% CI 1.90–3.06), suicidal ideation (OR 2.38; 95% CI 1.83–3.08) and attempts (OR 2.07; 95% CI 1.27–3.38), smoking tobacco cigarette (OR 1.96; 95% CI 1.45–2.65), cannabis use (OR 1.82; 95% CI 1.32–2.51) and binge drinking (OR 1.44; 95% CI 1.03–2.02). Cyberbullying victimisation was generally more strongly associated with mental health problems than substance use outcomes. Results were unchanged in sensitivity analyses that excluded participants who reported not using the internet (*N*  =  297), and those treating the independent variable (i.e., cyberbullying victimisation) as an ordered variable (four levels: never, once, two to three times, four or more times) showed that cyberbullying victimisation was associated with all the outcomes in a dose-fashion model, except for binge drinking. Parent–child relationship was a significant moderator of the association between cyberbullying victimisation and psychological distress (OR 2.19; 95% CI 1.06–4.51). More specifically, victims of cyberbullying who had negative relationship with their parents were more likely to experience psychological distress than their counterparts who reported a positive parent–child relationship. The relationship of cyberbullying victimisation with psychological distress and cannabis use significantly varied between males and females. Female adolescents who were victims of cyberbullying had greater odds of psychological distress (OR 2.69; 95% CI 1.46–4.98), suicidal ideation (OR 1.96; 95% CI 1.03–3.71) and cannabis use (OR 1.95; 95% CI 1.20–3.17) than their male counterparts. Results of logistic regression analyses examining the association between cyberbullying victimisation and psychological distress stratified by parent–child relationship and child's sex are outlined in online Supplement Table S1. Victims of cyberbullying who reported a negative parent–child relationship had greater odds of psychological distress compared with those who reported a positive parent–child relationship. Furthermore, female adolescents, but not males who are victims of cyberbullying have greater odds of psychological distress.

**Table 4.** Crude and adjusted ORs for the associations between cyberbullying victimisation and mental health and substance use outcomes among adolescents, OSDUHS, 2015

	Self-rated poor mental health	Psychological distress	Suicidal ideation	Suicide attempt	Smoking tobacco cigarette	Cannabis	Binge drinking
	OR (95% CI)	OR (95% CI)	OR (95% CI)	OR (95% CI)	OR (95% CI)	OR (95% CI)	OR (95% CI)
Model 1	**3.30 (2.60–4.17)** (*N* = 5295)	**3.63 (2.87–4.60)** (*N* = 5391)	**3.99 (3.26–4.89)** (*N* = 5188)	**4.46 (2.65–7.52)** (*N* = 5197)	**1.90 (1.46–2.47)** (*N* = 5383)	**1.58 (1.26–1.99)** (*N* = 5391)	1.21 (0.93–1.56) (*N* = 5371)
Model 2	**2.15 (1.64–2.81)** (*N* = 5074)	**2.41 (1.90–3.06)** (*N* = 5160)	**2.38 (1.83–3.08)** (*N* = 4991)	**2.07 (1.27–3.38)** (*N* = 4999)	**1.96 (1.45–2.65)** (*N* = 5153)	**1.82 (1.32–2.51)** (*N* = 5160)	**1.44 (1.03–2.02)** (*N* = 5143)
Model 3 Cyberbullying × parent–child relationship	0.79 (0.37–1.69) (*N* = 5067)	**2.19 (1.06–4.51)** (*N* = 5153)	0.88 (0.39–1.99) (*N* = 4984)	0.51 (0.16–1.60) (*N* = 4992)	0.76 (0.39–1.48) (*N* = 5149)	0.66 (0.35–1.26) (*N* = 5153)	0.53 (0.25–1.14) (*N* = 5136)
Model 4 Cyberbullying × sex	1.18 (0.69–2.01) (*N* = 5074)	**2.69 (1.46–4.98)** (*N* = 5160)	**1.96 (1.03–3.71)** (*N* = 4991)	0.73 (0.24–2.27) (*N* = 4999)	1.31 (0.71–2.39) (*N* = 5153)	**1.95 (1.20–3.17)** (*N* = 5160)	1.46 (0.80–2.69) (*N* = 5143)

OR, odds ratio; CI, confidence interval.

Model 1 is unadjusted.

Model 2 is adjusted for age, sex, ethnicity, subjective socioeconomic status and involvement in physical fighting, bullying victimisation and perpetration at school.

Model 3 is full model for each outcome + interaction term between Cyberbullying and parent–child relationship.

Model 4 is full model for each outcome + interaction term between Cyberbullying and sex.

Bold values represent ORs that are statistically significant at *a* = 0.05.

The three-way interaction term (cyberbullying victimisation × child's sex × parent–child relationship) was significant for the psychological distress model. After stratification by child's sex and parent–child relationship (Table 5), the adjusted model indicates that the association between cyberbullying victimisation and psychological distress was much stronger among males who have a negative relationship with their parents. However, cyberbullying victimisation was associated with greater odds of psychological distress among females regardless the nature of the relationship with their parents. There were evident sex differences in odds of psychological distress among victims of cyberbullying who reported positive parent–child relationship, but not negative relationship. The strength of the association between cyberbullying victimisation and psychological distress among adolescents who reported negative parent–child relationship was stronger among males than females. A sensitivity analysis excluding participants who reported no mother or no father (*N*  =  245) showed similar results.

**Table 5.** Crude and adjusted ORs for the association between cyberbullying victimisation and psychological distress stratified by sex and by getting along with parents, OSDUHS, 2015

	Psychological distress	
	Males	Females
	OR (95% CI)	OR (95% CI)
**Parent–child relationship**		
Positive		
Model 1	1.80 (1.01–3.22) (*N* = 2135)	4.28 (3.01–6.08) (*N* = 2537)
Model 2	1.17 (0.66–2.05) (*N* = 2042)	3.70 (2.46–5.57) (*N* = 2430)
**Parent–child relationship**		
Negative		
Model 1	7.94 (3.10–20.28) (*N* = 275)	4.89 (1.95–12.26) (*N* = 437)
Model 2	6.72 (2.65–17.01) (*N* = 259)	2.64 (1.14–6.11) (*N* = 422)

OR, odds ratio; CI, confidence interval.

Model 1 is unadjusted.

Model 2 is adjusted for age, ethnicity, subjective socioeconomic status and involvement in physical fighting, bullying victimisation and perpetration at school.

## Discussion

This large population-based study showed the existence of strong associations between cyberbullying victimisation and psychological distress, poor self-rated mental health, suicidal ideation and attempts, smoking tobacco cigarette, cannabis use and binge drinking after adjusting for important covariates. The association between cyberbullying victimisation and psychological distress was moderated by parent–child relationship and child's sex. Among females, cyberbullying victimisation was associated with psychological distress regardless of the strength of their relationship with their parents, while in males, it is those who had negative relationship with their parents who had much stronger association between cyberbullying victimisation and experiences of psychological distress.

Our findings are in line with previous studies that have shown that positive parent–child relationships have a protective effect on negative adolescent outcomes (Aseltine *et al*., 1998; Gribble Patricia *et al*., 2006; O'Brien and Mosco, 2012; Qu *et al*., 2015; Stafford *et al*., 2016). Elgar *et al*. (2014) showed that family dinners – seen as an opportunity for family contact and communication – moderate the associations between cyberbullying and internalising, externalising and substance use problems among students aged 12–18 years. However, our study extends previous findings by using a more direct measure of the parent–child relationship and by indicating that the relationship between cyberbullying victimisation and internalising and externalising behaviour vary by both parent–child relationship and sex.

A large body of research has demonstrated that females are particularly vulnerable to experiencing distress and depressive symptoms from early adolescence through adulthood (Kessler *et al*., 1993; Nolen-Hoeksema, 1995). Females tend to place more importance on peer relationships and on interpersonal experiences (Cyranowski *et al*., 2000), and are more likely to ruminate when faced with interpersonal stress or other difficult life events (Mezulis *et al*., 2002). Engaging in rumination may replace more adaptive coping strategies such as problem-solving and behavioural activation (Ward *et al*., 2003), and can maintain or exacerbate distress and depression (Nolen-Hoeksema *et al*., 2008), particularly among females (Broderick and Korteland, 2004; Burwell and Shirk, 2007; Abela and Hankin, 2011). In a prospective study, it was shown that rumination mediated the association between cyber-victimisation and depressive symptoms for the females, but not males (Feinstein *et al*., 2014).

Previous research has drawn attention to sex differences in the nature and buffering effect of parent–child relationship (Borawski *et al*., 2003; Hawkins *et al*., 2006). Parent–child relationships differ by sex of both parent and child, with mother–daughter relationships being generally stronger than father–daughter relationships (Steinberg, 1987), and fathers being usually closer to their sons than daughters (Starrels, 1994). However, the reasons as to why parent–child relationship may have different effects on adolescent males and females are very complex, as they may depend on a wide range of parameters, including children and parents (e.g., age, education, employment) characteristics, family structure and parenting styles (e.g., authoritativeness, discipline, nurturance). Because girls place more importance on their peer relationships, even a strong parental attachment might not be able to counteract the vulnerability factors of rumination and interpersonal distress. Providing parents with psycho-education may reduce depression by reducing maladaptive coping strategies such as rumination (Gate *et al*., 2013).

Even though males were more likely than females to report cannabis use, it is females who were victims of cyberbullying who had greater odds of cannabis use. These paradoxical findings suggest that female victims of cyberbullying may be more prone to cannabis use when facing negative experiences, such as the experience of cyberbullying victimisation. It may also be the case that females who are in distress and are ruminating about online attacks are more likely to make poor coping decisions by using cannabis (Nolen-Hoeksema *et al*., 2007; Aldao *et al*., 2010). It is also possible that females who use cannabis constitute an ideal target for cyberbullying victimisation. As such, cannabis use may represent important risk factors or behavioural marker for cyberbullying victimisation among adolescents. Longitudinal studies have documented both possibilities (Maniglio, 2015). For example, Gamez-Guadix *et al*. (2013) found that substance use predicted cyberbullying victimisation, but the latter did not predict substance use. Earnshaw *et al*. (2017) have recently showed that experiences of peer victimisation in early adolescence may have long-term effects on substance use behaviours during mid- to late-adolescence. Addressing cannabis use among female middle and high school students may help reduce, at least in part the prevalence of cyberbullying victimisation.

This study has several strengths and limitations worth mentioning. Important strengths include the use of a large and representative sample of middle and high school students across Ontario, and a comprehensive set of covariates. However, our results need to be interpreted considering the following limitations. First, the cross-sectional nature of the data precludes inferences about causality or temporality. It is possible that adolescents who exhibit externalising and internalising behaviours constitute a target of cyberbullies (Gamez-Guadix *et al*., 2013). Second, the data are based on self-report and may, thus, be subject to recall and desirability bias, especially for more sensitive questions, such as those related to mental health and cyberbullying victimisation. Third, the survey did not have questions on other forms of cyberbullying victimisation, such as those occurring via text messaging. Future studies are needed to capture this information. Another limitation of our study is related to the use of single items to assess the nature of parent–child relationship, involvement in bullying behaviour and self-rated mental health, which may raise potential issues related to reliability. Future studies using more refined tools are desired.

Despite limitations, these findings suggest that positive parent–child relationship may buffer the effect of cyberbullying victimisation on the risk of psychological distress among adolescent males. Our results have several important implications. With the rapid advances in information communication and technology and increasing popularity of social networking sites, there is a crucial need for more efficient strategies to address cyberbullying and related mental health problems (Sampasa-Kanyinga and Hamilton, 2015*a*, 2015*b*; Sampasa-Kanyinga and Lewis, 2015). Our results also support the need for intervention programmes to foster strong parent–child relationships, through education and promotion of good parenting practices. Increasing parental awareness about the buffering effect of the parent–child relationship may help them evaluate and enhance relationships with their children. Our results also provide further support for the importance of screening for cyberbullying victimisation in mental health settings. Mental health professionals could adopt a family centred approach for supporting adolescents who are victims of cyberbullying.

## Supplementary material

For supplementary material accompanying this paper visit http://dx.doi.org/10.1017/S2045796018000653. click here to view supplementary material

## Availability of data and materials

The OSDUHS data cannot be made available in the manuscript, the supplemental files or a public repository due to ethical and data management requirements. Researchers may directly contact the Centre for Addiction and Mental Health at info@camh.ca.
