# Supplementary material for: Cyberbullying victimisation and internalising and externalising problems among adolescents: the moderating role of parent–child relationship and child's sex
Source: Epidemiol Psychiatr Sci. 2018 Nov 13;29:e8. doi: 10.1017/S2045796018000653 (PMC8061125; doi:10.1017/S2045796018000653)
Supplement: Supplementary file 1 [file S2045796018000653sup001.docx]

|  | Parent-child relationship | | Child’s sex | |
| --- | --- | --- | --- | --- |
|  | Positive  (N=4,472) | Negative  (N=681) | Males  (N=2,307) | Females  (N=2,853) |
|  | aOR (95% CI) | aOR (95% CI) | aOR (95% CI) | aOR (95% CI) |
| Cyberbullying victimization |  |  |  |  |
| No | 1 | 1 | 1 | 1 |
| Yes | 2.27 (1.72 – 3.01) | 3.79 (1.87 – 7.67) | 1.42 (0.93 – 2.17) | 3.60 (2.46 – 5.27) |
| Age | 1.22 (1.14 – 1.30) | 1.18 (1.00 – 1.38) | 1.28 (1.15 – 1.42) | 1.22 (1.12 – 1.32) |
| Sex |  |  |  |  |
| Males | 1 | 1 |  |  |
| Females | 2.82 (2.28 – 3.48) | 2.70 (1.62 – 4.49) |  |  |
| Ethnicity |  |  |  |  |
| White | 1 | 1 | 1 | 1 |
| Black | 0.93 (0.55 – 1.57) | 1.08 (0.43 – 2.70) | 0.85 (0.37 – 1.98) | 1.07 (0.66 – 1.74) |
| East/SES Asian | 1.26 (0.77 – 2.06) | 2.00 (0.88 – 4.53) | 1.22 (0.65 – 2.26) | 1.43 (0.87 – 2.34) |
| South Asian | 1.56 (1.08 – 2.24) | 2.14 (1.15 – 3.97) | 1.70 (0.97 – 2.97) | 1.39 (0.92 – 2.08) |
| Other | 0.99 (0.68 – 1.43) | 2.51 (1.28 – 4.92) | 0.91 (0.52 – 1.58) | 1.60 (1.10 – 2.32) |
| Subjective socioeconomic status |  |  |  |  |
| Low | 1 | 1 | 1 | 1 |
| High | 0.55 (0.44 – 0.69) | 0.61 (0.36 – 1.03) | 0.41 (0.28 – 0.61) | 0.61 (0.44 – 0.83) |
| Physical fighting | 1.15 (0.96 – 1.37) | 1.02 (0.81 – 1.29) | 1.12 (0.96 – 1.31) | 1.34 (0.97 – 1.84) |
| School bullying victimization | 1.42 (1.26 – 1.61) | 1.58 (1.23 – 2.02) | 1.58 (1.39 – 1.79) | 1.35 (1.16 – 1.56) |
| School bullying perpetration | 1.08 (0.87 – 1.33) | 1.13 (0.88 – 1.47) | 1.10 (0.87 – 1.39) | 1.26 (0.97– 1.65) |

**Table S1.** Associations between cyberbullying victimization and psychological distress stratified by parent-child relationship and child’s sex, OSDUHS, 2015

aOR: adjusted odds ratio.

Models are adjusted age, sex, ethnicity, subjective socioeconomic status, and involvement in physical fighting, bullying victimization and perpetration at school for parent-child relationship -related models and for age, ethnicity, subjective socioeconomic status, and involvement in physical fighting, bullying victimization and perpetration at school for Child’s sex-related models.
